# Patient-Perceived Chronic Illness Care and Its Association with Health-Related Quality of Life Among Community-Dwelling Older Adults with Multimorbidity

**DOI:** 10.3390/healthcare14193243

**Published:** 2026-10-01

**Authors:** Lin Xia, Xiaolong Wang, Yuting Yang, Yulong Ma, Mingxia Jing

**Affiliations:** School of Public Health, Shihezi University, Bei’er Road, Shihezi 832003, China; xialin@stu.shzu.edu.cn (L.X.); wangxiaolong@stu.shzu.edu.cn (X.W.); yangyuting@stu.shzu.edu.cn (Y.Y.)

**Keywords:** multimorbidity, older adults, Patient Assessment of Chronic Illness Care, health-related quality of life, primary healthcare services

## Abstract

**Objective**: This study aims to assess how community-dwelling older adults with multimorbidity perceive chronic illness care, examine its association with health-related quality of life (HRQoL), and identify the management dimensions most closely associated with HRQoL. These findings may provide evidence for optimizing patient-centered multimorbidity management in primary care settings. **Methods**: This cross-sectional study involved 263 older adults with multimorbidity managed by primary healthcare institutions within a division of the Xinjiang Production and Construction Corps, China, from July to November 2024. The Patient Assessment of Chronic Illness Care (PACIC), which includes five care-process dimensions, was used to evaluate patient-perceived chronic illness care. HRQoL was measured using the EuroQol five-dimensional five-level questionnaire (EQ-5D-5L). Multiple linear regression identified factors associated with PACIC scores, while Spearman correlation and two-limit Tobit regression models examined the associations between PACIC scores and health utility values. **Results**: The mean overall PACIC score was 2.24 ± 0.27, and follow-up/coordination had the lowest dimension score (1.95 ± 0.48). Rural residence, living alone, polypharmacy, and activities of daily living impairment were independently associated with lower PACIC scores. After adjustment for prespecified covariates, a one-point higher overall PACIC score was associated with an estimated 0.232 higher EQ-5D-5L health utility value (95% CI: 0.123, 0.342, *p* < 0.001). Among the five PACIC dimensions, only problem solving/contextual counseling (AME = 0.084, *p* = 0.005) and follow-up/coordination (AME = 0.094, *p* = 0.017) remained independently associated with health utility values. **Conclusions**: Community-dwelling older adults with multimorbidity reported relatively low levels of chronic illness care, particularly in follow-up and coordination. Patient-perceived chronic illness care was positively associated with HRQoL. Specifically, problem solving/contextual counseling and follow-up/coordination were the two dimensions that remained independently associated with health utility values. These findings offer empirical evidence for primary healthcare institutions to identify patient-perceived service gaps, prioritize patient-centered multimorbidity care, and enhance health-service responsiveness.

## 1. Introduction

With the rapid aging of the population and the rising incidence of chronic diseases, multimorbidity has become increasingly prevalent among older adults. Defined as the presence of two or more chronic conditions in a single individual [1], multimorbidity affects over 50% of those aged 60 and above worldwide [2]. In China, the impact of multimorbidity on older adults is similarly significant [3,4]. This condition not only escalates healthcare usage and financial strain [5] but is also linked to diminished health-related quality of life (HRQoL), functional decline, and a higher risk of mortality [6,7]. Consequently, enhancing the quality of care and health outcomes for older adults with multimorbidity has emerged as a critical public health priority.

Older adults with multimorbidity often encounter significant challenges due to polypharmacy, frequent specialist consultations, and the demands of long-term self-management. Consequently, their care necessitates continuous, coordinated, and patient-centered integrated management [8]. Despite this need, current evaluations of multimorbidity care predominantly focus on healthcare providers’ perspectives or rely on disease-specific indicators [9], which may not fully capture patients’ actual experiences with care transitions, coordination, and self-management support. Evaluations from the patient perspective can directly reflect patients’ perceived care experiences and feedback, thereby highlighting service gaps that conventional evaluation methods might overlook [10]. Research indicates that integrating patient-reported care experiences and health outcomes into quality assessments is crucial for developing effective health service systems tailored to the needs of individuals with multimorbidity [11]. Utilizing these measures together can also pinpoint critical weaknesses in chronic disease management from the patient’s viewpoint, offering evidence to enhance health system responsiveness [12].

The ultimate aim of disease management is to improve health outcomes [13]. Health-Related Quality of Life (HRQoL) provides a comprehensive measure of patients’ physical functioning, psychological well-being, and daily activity limitations, making it a crucial indicator for assessing the health status of patients with multimorbidity [14]. For older adults facing multiple chronic conditions, health improvement hinges not only on managing disease-specific indicators but also on ensuring continuity, coordination, and personalization of care [8]. Thus, it is essential to evaluate chronic disease management services from the perspective of patients’ perceived care and explore their relationship with HRQoL. The Chronic Care Model (CCM) advocates for proactive, coordinated, and patient-centered care [15]. The Patient Assessment of Chronic Illness Care (PACIC), developed based on the CCM, is a patient-reported tool designed to measure how well the chronic illness care received aligns with CCM principles [16]. PACIC encompasses five dimensions: patient activation, delivery system design/decision support, goal setting/tailoring, problem solving/contextual counseling, and follow-up/coordination. By focusing on patients’ overall care experiences during long-term chronic disease management, PACIC is particularly suitable for evaluating perceived chronic illness care among older adults with multimorbidity [17].

The PACIC is extensively utilized to assess disease management services for individual chronic conditions like diabetes and hypertension, focusing on patients’ perceived service levels, dimension-specific differences, and related factors. Several studies have explored the link between PACIC scores and health outcomes. For instance, Schmittdiel et al. identified a positive correlation between PACIC scores and quality of life in 4108 patients with chronic conditions (OR = 1.20, 95% CI: 1.13, 1.27) [18]. Similarly, Akan et al. found PACIC scores to be significant predictors of all quality of life dimensions in patients with type 2 diabetes (*β* = 0.31–0.50) [19]. Zhou et al. demonstrated a significant association between PACIC scores and treatment adherence in patients with hypertension (*β* = −0.24) [20]. Despite evidence linking PACIC scores to health-related outcomes like quality of life and treatment adherence, most research has concentrated on single-disease or general chronic disease populations. There is limited evidence concerning older adults with multimorbidity. Notably, prior studies have primarily focused on the overall relationship between total PACIC scores and HRQoL, leaving the connections between specific PACIC dimensions and health utility insufficiently examined. Consequently, it remains unclear which PACIC dimensions are most strongly linked to HRQoL among older adults with multimorbidity.

**Figure 1:**
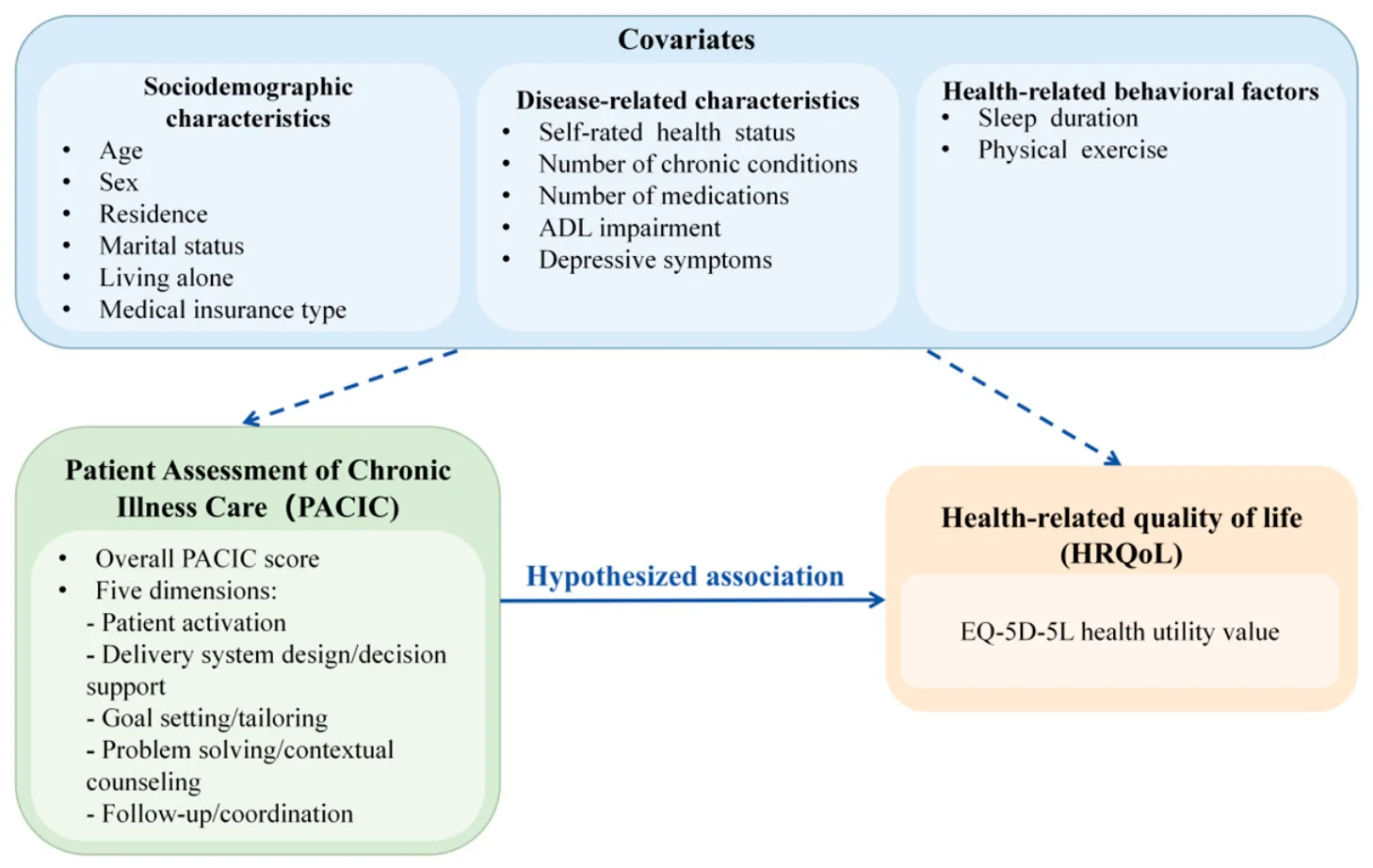
Conceptual framework for examining the association between patient-perceived chronic illness care and health-related quality of life among community-dwelling older adults with multimorbidity. Note: PACIC, Patient Assessment of Chronic Illness Care; HRQoL, health-related quality of life; EQ-5D-5L, EuroQol five-dimensional five-level questionnaire; ADL, activities of daily living. Solid arrow indicates the hypothesized association between PACIC and HRQoL. Dashed arrows represent potential confounding effects of covariates. All arrows indicate hypothesized associations and do not imply causality.

In China, primary healthcare institutions primarily manage chronic diseases, with recent policies integrating the management of multiple chronic conditions into basic public health services [21]. However, current multimorbidity management in primary care often relies on single-disease frameworks, which may not adequately address the complex needs of patients with multiple chronic conditions. The Xinjiang Production and Construction Corps, now experiencing an aging population, reported that individuals aged 60 and older constituted 12.78% of its population in 2020 [22]. Given the increased risk of multimorbidity with age [23], there is a heightened demand for effective management among older adults in this region. The Corps has developed a primary healthcare network centered on rural hospitals and community health service centers, offering services such as resident health record management, chronic disease follow-up, health education, and family doctor contracts. This infrastructure provides a solid foundation for assessing patient-perceived chronic illness care and its impact on health outcomes. In this context, our study focused on older adults with multimorbidity living in a division of the Xinjiang Production and Construction Corps. We utilized the PACIC to evaluate their perceived chronic illness care and identify influencing factors. Additionally, we employed EQ-5D-5L health utility values to measure HRQoL and examined the relationships between the total PACIC score, its five dimensions, and HRQoL. The study aimed to pinpoint key management dimensions more closely linked to health outcomes, which could be prioritized for service improvement. This would provide empirical evidence for optimizing patient-centered multimorbidity management and enhancing service responsiveness in primary care. The conceptual framework guiding this study, illustrating hypothesized associations among study variables, is depicted in Figure 1.

## 2. Materials and Methods

### 2.1. Study Design and Participants

This analytical cross-sectional study focused on older adults with multimorbidity, registered in health records and managed by primary healthcare institutions within a division of the Xinjiang Production and Construction Corps, China, from July to November 2024. To address potential differences in primary healthcare service availability, chronic disease management practices, and healthcare resource allocation between urban and rural areas, a stratified convenience sampling method was employed. This approach aimed to enhance the diversity of the study sample by including participants from varied primary healthcare settings.

Initially, study sites were categorized into urban and rural communities. Within each category, primary healthcare institutions were chosen based on several criteria: (1) the availability of chronic disease management services; (2) the capacity to identify eligible community-dwelling older adults with multimorbidity using existing health records; and (3) the feasibility of standardized data collection and on-site cooperation. Ultimately, five survey sites were selected, comprising three urban and two rural communities. At each site, eligible older adults with multimorbidity were recruited from those registered in health records, adhering to specific inclusion and exclusion criteria. Face-to-face questionnaire surveys were then conducted with these participants.

The study’s inclusion criteria required participants to be aged 60 years or older, have resided continuously at the selected survey site for at least six months, and possess a physician-confirmed diagnosis of two or more chronic diseases. The chronic conditions considered in this study encompassed 12 categories: hypertension, diabetes mellitus, coronary heart disease, stroke, dyslipidemia, malignant tumors, chronic pulmonary disease, liver disease, kidney disease, gastric or other digestive system diseases, arthritis or rheumatic disease, and asthma. Exclusion criteria included severe hearing impairment or communication difficulties that hindered questionnaire completion, as well as severe cognitive impairment or dementia that impeded understanding of the survey content. The Ethics Committee of the First Affiliated Hospital of Shihezi University approved this study (approval number: KJ2024-210-01, approval date: 28 June 2024). All participants provided written informed consent.

This analytical cross-sectional study, exploratory in nature, did not include a formal a priori power calculation for Tobit regression. Consequently, the sample size was determined by the number of eligible participants available during the study period. Ultimately, 264 participants were recruited from five survey sites, with 263 participants providing complete core variable data for the final analysis.

### 2.2. Variables and Measurements

Data were collected using a structured questionnaire comprising a researcher-developed general information section and four previously published standardized instruments: the Patient Assessment of Chronic Illness Care (PACIC), the EuroQol five-dimensional five-level questionnaire (EQ-5D-5L), the Patient Health Questionnaire-9 (PHQ-9), and the Activities of Daily Living (ADL) scale. The general information section collected sociodemographic characteristics, disease-related factors, and health-related behaviors. Sociodemographic variables included age, sex, residence, marital status, living arrangement, and type of medical insurance. Disease-related variables included self-rated health status, number of chronic conditions, and number of medications, while health-related behaviors included sleep duration and physical exercise. Depressive symptoms and ADL impairment were assessed using the PHQ-9 and ADL scale, respectively. Physical exercise was recorded as a binary variable (yes/no). Self-reported sleep duration was categorized as ≤6 h or >6 h. The number of long-term medications was categorized as <5 or ≥5, and the number of chronic conditions was grouped as 2–3, 4–5, or ≥6 based on the 12 physician-diagnosed chronic disease categories described above.

The Patient Assessment of Chronic Illness Care (PACIC) was utilized to evaluate patient-perceived chronic illness care. Developed by Glasgow et al. in 2005, the PACIC is grounded in the Chronic Care Model (CCM) and focuses on assessing chronic illness care from the patient’s viewpoint [16]. This scale comprises 20 items distributed across five dimensions: patient activation, delivery system design/decision support, goal setting/tailoring, problem solving/contextual counseling, and follow-up/coordination. Each item is scored on a 5-point Likert scale, where 1 signifies “never”, and 5 signifies “always.” Dimension scores are calculated as the average of the items within each dimension, while the overall PACIC score is the mean of all 20 items. Higher scores, both overall and within dimensions, suggest that the care perceived by patients aligns more closely with patient-centered chronic illness care principles. In this study, the Chinese version of the PACIC was employed. Its structure, dimensions, and scoring methods were consistent with previous applications in Chinese primary healthcare settings. The Chinese PACIC has shown acceptable reliability and validity among patients with chronic diseases. The questionnaire was administered without any modifications; no items were added, removed, or significantly altered [24].

Health-related quality of life was assessed using the EuroQol five-dimensional five-level questionnaire (EQ-5D-5L). This standardized tool measures health-related quality of life (HRQoL) through two main components: the EQ-5D descriptive system and the visual analogue scale (VAS) [25]. The EQ-5D descriptive system evaluates five dimensions: mobility, self-care, usual activities, pain/discomfort, and anxiety/depression. Each dimension is rated on a five-point scale, from 1 (“no problems”) to 5 (“extreme problems/unable to”), offering a detailed overview of an individual’s health status. In this study, we utilized the EQ-5D descriptive system and calculated health utility values using the Chinese EQ-5D-5L value set [26]. These utility values range from −0.391 to 1.000, with higher values indicating better health status. The EQ-VAS asks respondents to rate their perceived health on a scale from 0 to 100. However, due to potential comprehension difficulties among older participants and the influence of educational level on VAS scores [27], EQ-VAS scores were excluded from this study.

Depressive symptoms were evaluated using the Patient Health Questionnaire-9 (PHQ-9), which comprises nine items. Each item is scored on a scale from 0 to 3, where 0 means “not at all,” 1 indicates “several days,” 2 represents “more than half the days,” and 3 signifies “nearly every day.” The total score can range from 0 to 27. Scores between 0 and 4 suggest the absence of depressive symptoms, while a score of 5 or higher indicates a positive screening for depressive symptoms [28]. The PHQ-9 has shown strong reliability and validity among older adults in China, evidenced by a Cronbach’s *α* of 0.83 [29].

The assessment of daily living activities was conducted using the Activities of Daily Living (ADL) scale, which is divided into two subscales: the Physical Self-Maintenance Scale (PSMS) and the Instrumental Activities of Daily Living Scale (IADL), together comprising 14 items [30]. The PSMS evaluates six activities: eating, toileting, dressing, grooming, walking, and bathing. Meanwhile, the IADL assesses eight activities: food preparation, shopping, telephone use, housekeeping, laundry, use of transportation, medication use, and financial management. Each activity is rated on a 4-point scale: 1 indicates no difficulty, 2 some difficulty, 3 great difficulty, and 4 an inability to complete the task. The total score ranges from 14 to 56, with higher scores reflecting poorer ADL function. A score of 1 for any item signifies normal function, while scores between 2 and 4 suggest functional impairment. A total ADL score of 14 denotes normal ADL function, whereas any score above 14 indicates impairment.

### 2.3. Data Collection and Quality Control

Data collection was conducted through on-site, face-to-face questionnaire surveys. Prior to this, the research team delivered standardized training to all investigators, ensuring they comprehended the study’s objectives, survey procedures, questionnaire completion requirements, and essential quality control measures. During the data collection phase, investigators clearly communicated the study’s purpose, provided instructions for completing the questionnaire, and outlined relevant precautions to each participant. Upon receiving informed consent, investigators offered personalized guidance to assist participants in completing the questionnaire.

All questionnaires were collected on-site and immediately reviewed for completeness and logical consistency. Two trained researchers independently entered the data using a double-entry method, followed by cross-checking to ensure accuracy. Out of 264 completed and returned questionnaires, one was excluded due to missing core variables. This exclusion resulted in 263 participants being included in the final analysis, yielding a valid questionnaire rate of 99.6%.

### 2.4. Statistical Analysis

The data analysis was conducted using Stata version 16.0. For continuous variables with a normal distribution, results are reported as means with standard deviations. Non-normally distributed continuous variables are described using medians and interquartile ranges [Median (Q1, Q3)]. Categorical variables are expressed in terms of frequencies and percentages [*n* (%)]. In univariate analyses, normally distributed continuous variables were compared using the independent-samples *t*-test or analysis of variance. Conversely, non-normally distributed continuous variables were analyzed using the Mann–Whitney U test or Kruskal–Wallis H test, depending on the specific requirements.

To determine the factors influencing the overall PACIC score, multiple linear regression analysis was employed. Variables that showed a significance level of *p* < 0.05 in the univariate analyses were included in the multivariable model. Multicollinearity was evaluated using variance inflation factors (VIFs), where a VIF of less than 5 was deemed indicative of no significant multicollinearity [31].

Spearman’s rank correlation analysis was conducted to explore the relationships between the overall PACIC score, its five dimension scores, and the five EQ-5D-5L dimensions, as well as the EQ-5D-5L health utility value. Given that EQ-5D-5L health utility values range from −0.391 to 1.000 and exhibit significant clustering at the upper boundary, a two-limit Tobit regression was employed as the primary analytical method. The lower and upper limits were set at −0.391 and 1.000, respectively. This method presumes a censored-normal latent outcome distribution; thus, additional regression models were tested in sensitivity analyses to evaluate the robustness of the results. In Model 1, the overall PACIC score was the primary independent variable, while Model 2 included the five PACIC dimension scores to identify dimensions independently linked to health utility. Both models were adjusted for factors such as age, sex, place of residence, marital status, number of chronic conditions, number of medications, ADL impairment, and depressive symptoms. These covariates were chosen based on prior evidence and clinical significance [32,33]. To improve result interpretability, average marginal effects (AMEs) on the observed EQ-5D-5L health utility scale were calculated following the Tobit regression.

To evaluate the robustness of the main findings, four sensitivity analyses were conducted. The first analysis employed ordinary least squares (OLS) regression with robust standard errors on the full sample. In the second analysis, OLS with robust standard errors was repeated, excluding participants with an EQ-5D-5L health utility value of 1.000, to examine the potential impact of upper-bound clustering. Third, considering the multicenter sampling structure, an exploratory sensitivity analysis utilized survey-site cluster-robust standard errors. Due to the limited number of survey sites (five), these cluster-robust results were interpreted with caution and not deemed definitive inferential evidence. Lastly, the primary Tobit models were rerun without adjusting for ADL impairment to explore the potential effects of over-adjustment. Except for the analysis excluding ADL impairment, the same set of covariates as in the primary Tobit models was applied.

## 3. Results

### 3.1. Baseline Characteristics of Participants

A total of 263 community-dwelling older adults with multimorbidity were included in this study. The mean age of the participants was 78.58 ± 6.09 years. The mean number of chronic conditions was 4.02 ± 1.72, ranging from 2 to 10 conditions. Participants aged 80 years or older accounted for 60.5% of the sample (159/263), women accounted for 61.2% (161/263), residents of rural communities accounted for 52.1% (137/263), and participants with a spouse accounted for 66.5% (175/263). The baseline characteristics of the participants are presented in Table 1.

### 3.2. Univariate Analysis of PACIC Scores

The univariate analysis showed that the overall PACIC score differed significantly by age, place of residence, marital status, living arrangement, number of medications, and ADL impairment among older adults with multimorbidity (all *p* < 0.05). In contrast, no statistically significant differences in the overall PACIC score were observed according to sex, medical insurance type, self-rated health status, sleep duration, physical exercise, number of chronic conditions, or depressive symptoms (all *p* > 0.05). The PACIC scores and comparison results across participant characteristics are presented in Table 1.

### 3.3. Patient-Perceived Chronic Illness Care Among Community-Dwelling Older Adults with Multimorbidity

The overall PACIC score was 2.24 ± 0.27. Among the five dimensions, the scores, from highest to lowest, were: delivery system design/decision support (2.56 ± 0.51), patient activation (2.45 ± 0.55), problem solving/contextual counseling (2.27 ± 0.58), goal setting/tailoring (2.19 ± 0.61), and follow-up/coordination (1.95 ± 0.48). These scores are detailed in Table 2. The PACIC demonstrated acceptable internal consistency in this sample, with Cronbach’s α values ranging from 0.664 to 0.851 for the dimensions and 0.721 for the overall scale. Confirmatory factor analysis supported the five-factor model fit, as indicated by *χ*^2^/df = 2.06, CFI = 0.903, TLI = 0.920, RMSEA = 0.051, and SRMR = 0.073.

### 3.4. Factors Associated with the Overall PACIC Score

Variables that were statistically significant in the univariate analyses were included in the multiple linear regression model. The overall model was statistically significant (*F* = 13.86, *p* < 0.001), with an adjusted *R*^2^ of 0.256. Multicollinearity diagnostics showed that the VIF values for all independent variables were less than 5, with a mean VIF of 2.05, and all tolerance values were greater than 0.2, indicating no serious multicollinearity in the model (Table S1).

The multiple linear regression analysis identified that place of residence, living arrangement, number of medications, and ADL impairment were independently linked to the overall PACIC score (*p* < 0.05). While age was significant in the univariate analysis, it did not independently correlate with the PACIC score in the multivariable linear regression. Residents of rural communities scored lower on the overall PACIC compared to their urban counterparts (B = −0.151, *p* < 0.001). Participants not living alone had a higher overall PACIC score than those living alone (B = 0.168, *p* < 0.001). Additionally, individuals taking five or more medications had a lower PACIC score compared to those taking fewer than five (B = −0.078, *p* = 0.010). Participants without ADL impairment also scored higher on the PACIC than those with impairment (B = 0.097, *p* = 0.002). These findings are detailed in Table 3.

### 3.5. Correlations of PACIC Scores with EQ-5D-5L Dimensions and Health Utility Values

Spearman’s rank correlation analysis showed that the overall PACIC score, and all dimension scores were positively correlated with EQ-5D-5L health utility values (*r_s_* = 0.273–0.394, all *p* < 0.05), with the strongest correlation observed between the overall PACIC score and the health utility value (*r_s_* = 0.394, *p* < 0.05). The overall PACIC score and all dimension scores were negatively correlated with the five EQ-5D-5L dimensions (*r_s_* = −0.355 to −0.130, all *p* < 0.05). Since higher scores on the EQ-5D-5L dimensions indicate more severe health issues, these results suggest that participants perceiving higher levels of chronic illness care reported fewer issues in mobility, self-care, usual activities, pain/discomfort, and anxiety/depression. Detailed results can be found in Table 4.

### 3.6. Tobit Regression Analysis of Average Marginal Effects for the Association Between PACIC Scores and EQ-5D-5L Health Utility Values

The theoretical range of the EQ-5D-5L health utility value was −0.391 to 1.000. In this study, one participant (0.4%) reached the theoretical lower limit, and 77 participants (29.3%) reached the theoretical upper limit, indicating clustering of health utility values at the scale boundaries, primarily at the upper boundary. Therefore, a two-limit Tobit model was further used to examine the association between PACIC scores and health utility values. Multicollinearity diagnostics showed that the VIF values for the five PACIC dimensions ranged from 1.60 to 2.12, with a mean VIF of 1.79, indicating no serious multicollinearity among the dimensions (Table S2).

The analysis of average marginal effects, adjusted for factors such as age, sex, place of residence, marital status, number of chronic conditions, number of medications, ADL impairment, and depressive symptoms, revealed a positive association between the overall PACIC score and the health utility value. Specifically, a one-point increase in the overall PACIC score corresponded to an average rise of 0.232 in the EQ-5D-5L health utility value (95% CI: 0.123–0.342, *p* < 0.001). When examining the five PACIC dimensions together, problem solving/contextual counseling and follow-up/coordination emerged as independently and positively linked to the health utility value. A one-point increase in the problem solving/contextual counseling score resulted in an average increase of 0.084 in the health utility value (95% CI: 0.026–0.143, *p* = 0.005). Similarly, a one-point rise in the follow-up/coordination score led to an average increase of 0.094 (95% CI: 0.017–0.171, *p* = 0.017). In contrast, the dimensions of patient activation, delivery system design/decision support, and goal setting/tailoring did not show statistically significant associations with the health utility value (all *p* > 0.05). These findings are detailed in Table 5.

Sensitivity analyses employed four alternative model specifications: (1) OLS regression with robust standard errors on the full sample; (2) OLS regression with robust standard errors excluding participants with an EQ-5D-5L health utility value of 1.000; (3) two-limit Tobit regression with survey-site cluster-robust standard errors; and (4) two-limit Tobit regression without adjusting for ADL impairment. Across these models, the positive association between the overall PACIC score and EQ-5D-5L health utility values persisted. The associations for problem solving/contextual counseling and follow-up/coordination also remained generally consistent in direction and statistical significance, though some dimension-specific estimates varied. The primary Tobit coefficients and model fit statistics are detailed in Tables S3 and S4, while the sensitivity analyses are documented in Tables S5–S7.

## 4. Discussion

This study assessed how community-dwelling older adults with multimorbidity perceive chronic illness care and explored its link to health-related quality of life. The overall PACIC score was 2.24 ± 0.27, suggesting a relatively low level of patient-perceived chronic illness care compared to findings from previous studies on chronic disease populations [17,20,34,35]. This low score may be attributed to the increased complexity involved in managing multimorbidity. Older adults with multiple chronic conditions often face interacting and compounding disease burdens, polypharmacy, functional decline, and increased reliance on healthcare services [6,36,37,38]. Consequently, effective management of multimorbidity necessitates not only disease-specific interventions but also integrated, continuous, and individualized care strategies.

Among the five PACIC dimensions, delivery system design/decision support received the highest score, while follow-up/coordination scored the lowest. Similar deficiencies in follow-up and coordination have been noted in other primary care populations [20,35], indicating that continuity of care remains a widespread challenge despite standardized chronic disease management procedures. This issue is especially critical for older adults with multimorbidity, as their care often necessitates ongoing follow-up, medication management, information exchange, and coordination among healthcare providers [39,40,41]. Research involving older populations further indicates that improved continuity of care correlates with better chronic disease management, enhanced patient-reported experiences, and higher quality of life [42]. Therefore, enhancing follow-up and coordination should be a priority for advancing patient-centered multimorbidity care. Additionally, lower overall PACIC scores were observed among rural residents, individuals living alone, those experiencing polypharmacy, and those with ADL impairment. These findings imply that perceived deficiencies in chronic illness care are more prevalent among patients facing greater treatment complexity, functional limitations, or limited social and healthcare resources. From a service perspective, these characteristics can assist primary care providers in identifying patients who need more intensive follow-up and personalized support. Consequently, risk-stratified management that considers patients’ living situations, medication burdens, and functional statuses is particularly pertinent when allocating limited primary care resources.

This study further reveals that higher patient-perceived chronic illness care correlates independently with improved HRQoL. After adjusting for sociodemographic factors, disease burden, medication use, functional impairment, and depressive symptoms, each one-point increase in the overall PACIC score corresponds to an average marginal increase of 0.232 in the EQ-5D-5L health utility value. While this increase seems substantial compared to the commonly reported minimal important difference (MID) estimates for EQ-5D-5L utility values (approximately 0.03–0.05) [43], it should be considered within the context of the PACIC measurement structure. The PACIC consists of 20 items scored from 1 (“never”) to 5 (“always”), meaning a one-point increase signifies a significant enhancement across various dimensions of patient-perceived chronic care, rather than a minor incremental change. Thus, the observed association may suggest that comprehensive improvements in patients’ care experiences across multiple domains could lead to clinically meaningful differences in perceived health status. However, due to the study’s cross-sectional design, these findings should be viewed as associations rather than causal effects.

The PACIC encompasses five distinct dimensions of care processes. Notably, when all five dimensions were analyzed together using the Tobit model, only problem solving/contextual counseling and follow-up/coordination showed independent associations with health utility values. Specifically, each one-point increase in these dimensions corresponded to average marginal increases of 0.084 and 0.094, respectively. Problem solving/contextual counseling addresses care tailored to a patient’s unique circumstances, daily challenges, and treatment needs. In contrast, follow-up/coordination ensures continuity, facilitates information exchange, and integrates services across healthcare providers [16]. These processes are particularly pertinent for older adults with multimorbidity, who often encounter competing treatment priorities, significant treatment burdens, and fragmented care pathways [41,44,45]. The robustness of these associations across various sensitivity analyses underscores that responsive problem-solving support and continuous coordination are the PACIC components most strongly linked to HRQoL in this demographic. Consequently, these findings highlight specific care-process domains that merit further prospective evaluation as potential targets for quality-improvement interventions.

While patient activation, delivery system design/decision support, and goal setting/tailoring showed significant correlations with health utility values in unadjusted analyses, none remained independently associated after adjusting for other PACIC dimensions. This does not imply these components lack importance; rather, their influence on HRQoL might be indirect or conceptually overlap with other care processes. For instance, patient activation could enhance health outcomes by fostering better self-management behaviors, though the impact on HRQoL may differ based on patient characteristics and care settings [46]. Delivery system design/decision support is crucial for chronic illness care, yet its effectiveness in improving patient outcomes may hinge on its translation into personalized problem-solving and continuity of care [47]. Likewise, goal setting/tailoring might need ongoing review and integration into care processes to reveal its benefits, especially for older adults with multiple health conditions [48,49]. Thus, these dimensions likely serve as supportive elements within a broader coordinated care framework rather than acting as independent determinants of HRQoL.

These findings have significant implications for managing multimorbidity in primary care settings. While the results do not advocate for replacing standardized chronic disease management structures, they highlight the importance of supplementing these structures with enhanced individualized problem-solving support, as well as sustained follow-up and coordination. In resource-limited healthcare environments, the PACIC can be an effective tool for identifying patient groups with suboptimal care experiences and pinpointing specific care processes that require more attention. By integrating patient-reported care experiences with health utility measures, a practical framework can be established to identify service gaps, prioritize quality improvements, and enhance the responsiveness of primary care for older adults with multimorbidity.

## 5. Limitations

This study faces several limitations. Firstly, its cross-sectional design limits causal inference, necessitating cautious interpretation of the observed associations. Secondly, while stratified convenience sampling included participants from both urban and rural primary healthcare settings, selection bias cannot be excluded, restricting generalizability of the findings beyond the study region. Thirdly, despite adjusting for clinically relevant covariates based on prior evidence, residual confounding from unmeasured factors (e.g., disease severity, health literacy, social support, socioeconomic status, and healthcare utilization) cannot be entirely ruled out. Additionally, potential effect modification across key subgroups (e.g., age, sex, residence, and multimorbidity burden) was not explored. Although two-limit Tobit models were suitable for the bounded distribution of EQ-5D-5L utility values and multiple sensitivity analyses confirmed the robustness of the findings, formal a priori power calculation for the Tobit regression models was not conducted. Lastly, the use of self-reported measures may have introduced recall and reporting bias. Future multicenter longitudinal studies with larger and more diverse samples are necessary to clarify the relationship between PACIC and HRQoL and to further investigate whether these associations vary across different sociodemographic and clinical contexts.

## 6. Conclusions

This study revealed that older adults with multimorbidity perceive chronic illness care in primary care settings as relatively inadequate, particularly in follow-up and coordination, which scored lowest on the PACIC scale. A positive correlation was found between patient-perceived chronic illness care and health-related quality of life. Notably, problem solving/contextual counseling and follow-up/coordination exhibited the strongest independent associations with health utility values, indicating these areas as key targets for quality-improvement research. By aligning patient-reported care experiences with health outcomes, the study offers evidence for primary healthcare institutions to identify service gaps, enhance responsiveness, and optimize resource allocation efficiently. Future longitudinal and intervention studies are essential to ascertain whether enhancing proactive follow-up, coordinated management, and individualized problem-responsive support can improve care experiences and health-related quality of life for older adults with multimorbidity.

## Figures and Tables

**Table 1:** Baseline characteristics of participants and univariate analysis of PACIC scores (*n* = 263).

Variables and Categories	*n* (%)	PACIC Score, Mean ± SD	t/F	*p* Value
Age, years				
≤69	18 (6.8)	2.30 ± 0.25	5.85	0.003
70–79	86 (32.7)	2.31 ± 0.25		
≥80	159 (60.5)	2.19 ± 0.27		
Sex				
Female	161 (61.2)	2.21 ± 0.26	−1.89	0.060
Male	102 (38.8)	2.28 ± 0.28		
Residence				
Urban	126 (47.9)	2.32 ± 0.28	5.10	<0.001
Rural	137 (52.1)	2.16 ± 0.23		
Marital status				
Married	175 (66.5)	2.28 ± 0.27	3.52	<0.001
Unmarried/divorced/widowed	88 (33.5)	2.16 ± 0.24		
Living alone				
Yes	65 (24.7)	2.09 ± 0.21	−5.40	<0.001
No	198 (75.3)	2.29 ± 0.26		
Medical insurance type				
Publicly funded medical care	10 (3.8)	2.32 ± 0.31	0.67	0.513
Urban Employee Basic Medical Insurance	217 (82.5)	2.24 ± 0.27		
Urban and Rural Resident Basic Medical Insurance	36 (13.7)	2.21 ± 0.20		
Self-rated health status				
Poor	78 (29.7)	2.21 ± 0.23	0.56	0.574
Fair	98 (37.2)	2.25 ± 0.29		
Good	87 (33.1)	2.25 ± 0.27		
Sleep duration, h				
≤6	143 (54.4)	2.22 ± 0.24	−1.27	0.207
>6	120 (45.6)	2.26 ± 0.29		
Number of medications				
<5	125 (47.5)	2.31 ± 0.28	4.15	<0.001
≥5	138 (52.5)	2.18 ± 0.23		
Physical exercise				
Yes	211 (80.2)	2.25 ± 0.28	1.39	0.165
No	52 (19.8)	2.19 ± 0.16		
Number of chronic conditions				
2–3	112 (42.6)	2.26 ± 0.26	1.37	0.256
4–5	108 (41.1)	2.24 ± 0.30		
≥6	43 (16.3)	2.18 ± 0.19		
ADL impairment				
Yes	162 (61.6)	2.18 ± 0.26	−4.44	<0.001
No	101 (38.4)	2.33 ± 0.25		
Depressive symptoms				
Yes	112 (42.6)	2.20 ± 0.23	−1.76	0.080
No	151 (57.4)	2.26 ± 0.29		

Note: PACIC, Patient Assessment of Chronic Illness Care; ADL, activities of daily living; SD, standard deviation. Data are presented as *n* (%) or mean ± SD.

**Table 2:** Overall and dimension scores of the PACIC scale.

Dimension	Minimum	Maximum	Score, Mean ± SD
Patient activation	1	5	2.45 ± 0.55
Delivery system design/decision support	1	5	2.56 ± 0.51
Goal setting/tailoring	1	5	2.19 ± 0.61
Problem solving/contextual counseling	1	5	2.27 ± 0.58
Follow-up/coordination	1	5	1.95 ± 0.48
Overall PACIC score	1.7	3.9	2.24 ± 0.27

**Table 3:** Multiple linear regression analysis of factors associated with the overall PACIC score.

Variables and Categories	B	SE	Std. *β*	t	*p* Value	95% CI
Constant	2.214	0.071		31.18	<0.001	2.074, 2.354
Age, years						
≤69	Ref.					
70–79	0.016	0.061	0.029	0.27	0.791	−0.104, 0.136
≥80	−0.047	0.059	−0.087	−0.80	0.423	−0.163, 0.069
Residence						
Urban	Ref.					
Rural	−0.151	0.029	−0.284	−5.20	<0.001	−0.208, −0.094
Marital status						
Married	Ref.					
Unmarried/divorced/widowed	0.009	0.036	0.016	0.25	0.800	−0.061, 0.079
Living alone						
Yes	Ref.					
No	0.168	0.038	0.274	4.38	<0.001	0.093, 0.244
Number of medications						
<5	Ref.					
≥5	−0.078	0.030	−0.147	−2.58	0.010	−0.138, −0.019
ADL impairment						
Yes	Ref.					
No	0.097	0.031	0.178	3.11	0.002	0.036, 0.158

Note: PACIC, Patient Assessment of Chronic Illness Care; ADL, activities of daily living; SE, standard error; CI, confidence interval; Ref., reference category; B, unstandardized regression coefficient; Std. *β*, standardized regression coefficient. Model fit: *F* = 13.86, *p* < 0.001; adjusted *R*^2^ = 0.256. *p* < 0.05 was considered statistically significant.

**Table 4:** Spearman’s rank correlation analysis of PACIC scores with EQ-5D-5L dimensions and health utility values (*n* = 263).

Variable	Mobility	Self-Care	Usual Activities	Pain/Discomfort	Anxiety/Depression	Health Utility Value
Overall PACIC score	−0.308	−0.285	−0.355	−0.307	−0.205	0.394
Patient activation	−0.246	−0.244	−0.305	−0.233	−0.215	0.341
Delivery system design/decision support	−0.303	−0.287	−0.314	−0.280	−0.187	0.360
Goal setting/tailoring	−0.209	−0.206	−0.251	−0.229	−0.199	0.279
Problem solving/contextual counseling	−0.205	−0.231	−0.205	−0.280	−0.200	0.309
Follow-up/coordination	−0.233	−0.170	−0.219	−0.179	−0.130	0.273

Note: Values are Spearman’s rank correlation coefficients (*r_s_*). All correlations were statistically significant at *p* < 0.05. PACIC, Patient Assessment of Chronic Illness Care; EQ-5D-5L, EuroQol five-dimensional five-level questionnaire.

**Table 5:** Average marginal effects from Tobit regression for the associations of the overall PACIC score and dimension scores with EQ-5D-5L health utility values.

Model	Variable	AME	SE	*p* Value	95% CI
Model 1: Overall PACIC score	Overall PACIC score	0.232	0.056	<0.001	0.123, 0.342
Model 2: PACIC dimension scores	Patient activation	0.029	0.033	0.370	−0.035, 0.093
	Delivery system design/decision support	0.056	0.036	0.120	−0.015, 0.127
	Goal setting/tailoring	0.018	0.032	0.584	−0.045, 0.080
	Problem solving/contextual counseling	0.084	0.030	0.005	0.026, 0.143
	Follow-up/coordination	0.094	0.039	0.017	0.017, 0.171

Note: AME, average marginal effect; PACIC, Patient Assessment of Chronic Illness Care; EQ-5D-5L, EuroQol five-dimension five-level questionnaire; SE, standard error; CI, confidence interval. AMEs were estimated from Tobit models on the observed EQ-5D-5L health utility scale. Model 1 included the overall PACIC score, and Model 2 simultaneously included the five PACIC dimension scores. Both models were adjusted for age, sex, place of residence, marital status, number of chronic conditions, number of medications, ADL impairment, and depressive symptoms.

## Data Availability

The data supporting the findings of this study are available from the corresponding author upon reasonable request. The data are not publicly available due to privacy and confidentiality considerations.

## Supplementary Materials

The following supporting information can be downloaded at: https://www.mdpi.com/article/10.3390/healthcare14193243/s1, Table S1: Multicollinearity diagnostics for predictors included in the multiple linear regression model of the overall PACIC score; Table S2: Multicollinearity diagnostics for the five PACIC dimension scores included in the two-limit Tobit regression model; Table S3: Two-limit Tobit regression coefficients for the association between the overall PACIC score and EQ-5D-5L health utility values (*n* = 263); Table S4: Two-limit Tobit regression coefficients for the associations between the five PACIC dimension scores and EQ-5D-5L health utility values (*n* = 263); Table S5: Sensitivity analyses using OLS regression with robust standard errors in the full sample and after excluding participants with an EQ-5D-5L health utility value of 1.000; Table S6: Exploratory sensitivity analysis using survey-site cluster-robust standard errors for the associations between PACIC scores and EQ-5D-5L health utility values; Table S7: Sensitivity analysis excluding ADL impairment from covariate adjustment for the associations between PACIC scores and EQ-5D-5L health utility values.

## Abbreviations

The following abbreviations are used in this manuscript:

ADL	activities of daily living
AME	average marginal effect
CCM	Chronic Care Model
CI	confidence interval
EQ-5D	EuroQol five-dimensional descriptive system
EQ-5D-5L	EuroQol five-dimensional five-level questionnaire
EQ-VAS	EuroQol Visual Analogue Scale
HRQoL	health-related quality of life
IADL	Instrumental Activities of Daily Living
OLS	ordinary least squares
PACIC	Patient Assessment of Chronic Illness Care
PHQ-9	Patient Health Questionnaire-9
PSMS	Physical Self-Maintenance Scale
SD	standard deviation
SE	standard error
VAS	visual analogue scale
VIF	variance inflation factor
