# Supplementary material for: Patient-Perceived Chronic Illness Care and Its Association with Health-Related Quality of Life Among Community-Dwelling Older Adults with Multimorbidity
Source: Healthcare (Basel). 2026 Oct 1;14(19):3243. doi: 10.3390/healthcare14193243 (PMC13665224; doi:10.3390/healthcare14193243)
Supplement: Supplementary file 1 [file healthcare-14-03243-s001.zip › healthcare-4526136-supplementary.pdf]

Supplementary Materials

Table S1. Multicollinearity diagnostics for predictors included in the multiple linear regression model of the overall PACIC score

Variable	Variance inflation factor (VIF)	Tolerance (1/VIF)
Age group	-	-
70-79	4.1	0.244
≥ 80	4.14	0.241
Residence	1.05	0.955
Marital status	1.42	0.703
Living alone	1.37	0.729
Number of medications	1.14	0.874
ADL impairment	1.15	0.872
Mean VIF	2.05	—

Note: PACIC, Patient Assessment of Chronic Illness Care; VIF, variance inflation factor. All VIF values were < 5 (mean VIF = 2.05), indicating no evidence of serious multicollinearity among predictors included in the model.

Table S2. Multicollinearity diagnostics for the five PACIC dimension scores included in the two-limit Tobit regression model

Variable	Variance inflation factor (VIF)	Tolerance (1/VIF)
Patient activation	1.63	0.614
Delivery system design/decision support	1.90	0.528
Goal setting/tailoring	2.12	0.472
Problem solving/contextual counseling	1.60	0.625
Follow-up/coordination	1.73	0.579
Mean VIF	1.79	-

Note: PACIC, Patient Assessment of Chronic Illness Care; VIF, variance inflation factor. VIF values for the five PACIC dimension scores ranged from 1.60 to 2.12 (mean VIF = 1.79), indicating no evidence of serious multicollinearity.

Table S3. Two-limit Tobit regression coefficients for the association between the overall PACIC score and EQ-5D-5L health utility values (n = 263).

Variable	*B*	SE	*p* value	95% CI
Constant	-0.002	0.222	0.993	-0.440, 0.436
Overall PACIC score	0.359	0.087	< 0.001	0.188, 0.530
Age, years				
≤ 69	Ref.			
70-79	0.071	0.091	0.434	-0.107, 0.249
≥ 80	-0.010	0.087	0.910	-0.180, 0.161
Sex				
Female	Ref.			
Male	-0.028	0.044	0.526	-0.115, 0.059
Residence				
Urban	Ref.			
Rural	-0.019	0.044	0.671	-0.105, 0.068
Marital status				
Married	Ref.			
Unmarried/divorced/widowed	-0.034	0.047	0.471	-0.126, 0.059
Number of chronic conditions				
2–3	Ref.			
4–5	0.043	0.046	0.359	-0.049, 0.134
≥ 6	0.075	0.066	0.261	-0.056, 0.205
Number of medications				
< 5	Ref.			
≥ 5	-0.040	0.046	0.388	-0.130, 0.051
ADL impairment				
Yes	Ref.			
No	0.166	0.047	< 0.001	0.074, 0.259
Depressive symptoms				
Yes	Ref.			
No	0.018	0.043	0.683	-0.067, 0.102

Note: The model was adjusted for age, sex, place of residence, marital status, number of chronic conditions, number of medications, ADL impairment, and depressive symptoms. *B*, two-limit Tobit regression coefficient; SE, standard error; CI, confidence interval; Ref., reference category; PACIC, Patient Assessment of Chronic Illness Care; ADL, activities of daily living. Pseudo R^2 = 0.222; LR χ^2 = 60.13.

Table S4. Two-limit Tobit regression coefficients for the associations between the five PACIC dimension scores and EQ-5D-5L health utility values (n = 263).

Variable	*B*	SE	*p* value	95% CI
ıstant	-0.190	0.232	0.413	-0.648, 0.267
.ent activation	0.045	0.050	0.371	-0.054, 0.144
ivery system design/decision port	0.087	0.056	0.121	-0.023, 0.196
ıl setting/tailoring	0.027	0.049	0.584	-0.070, 0.125
blem solving/contextual nseling	0.130	0.046	0.005	0.039, 0.221
.ow-up/coordination	0.146	0.061	0.018	0.025, 0.267
;, years				
≤ 69	Ref.			
70- 79	0.082	0.090	0.363	-0.095, 0.259
≥ 80	0.001	0.086	0.996	-0.169, 0.170
Female	Ref.			
Male	-0.031	0.044	0.483	-0.118, 0.056
idence				
Urban	Ref.			
Rural	-0.016	0.044	0.722	-0.102, 0.071
:ital status				
Married	Ref.			
'nmarried/divorced/widowed	-0.031	0.047	0.511	-0.123, 0.061
nber of chronic conditions				
2-3	Ref.			
4-5	0.060	0.046	0.198	-0.031, 0.151
≥ 6	0.091	0.066	0.168	-0.039, 0.221
nber of medications				
< 5	Ref.			
≥ 5	-0.044	0.046	0.333	-0.135, 0.046
L impairment				
Yes	Ref.			
No	0.169	0.047	< 0.001	0.077, 0.261
›ressive symptoms				
Yes	Ref.			
No	0.022	0.043	0.604	-0.062, 0.106

Note: The model was adjusted for age, sex, place of residence, marital status, number of chronic conditions, number of medications, ADL impairment, and depressive symptoms. *B*, two-limit Tobit regression coefficient; SE, standard error; CI, confidence interval; Ref., reference category; PACIC, Patient Assessment of Chronic Illness Care; ADL, activities of daily living. Pseudo R^2 = 0.251; LR χ^2 = 67.95.

Table S5. Sensitivity analyses using OLS regression with robust standard errors in the full sample and after excluding participants with an EQ-5D-5L health utility value of 1.000

Model	Variable	OLS regression with robust standard errors, *B* (95% CI)	*p* value	OLS regression with robust standard errors after excluding participants with EQ-5D-5L utility = 1.000, *B* (95% CI)	*p* value
Model 1: Overall PACIC score	Overall PACIC score	0.256 (0.106, 0.406)	0.001	0.171 (0.010, 0.333)	0.038
Model 2: PACIC dimension scores	Patient activation	0.023 (-0.047, 0.093)	0.518	-0.046 (-0.145, 0.053)	0.361
	Delivery system design/decision support	0.043 (-0.026, 0.113)	0.221	0.014 (-0.046, 0.075)	0.643
	Goal setting/tailoring	0.039 (-0.015, 0.093)	0.160	0.045 (-0.014, 0.105)	0.135
	Problem solving/contextual counseling	0.090 (0.036, 0.144)	0.001	0.094 (0.036, 0.152)	0.002
	Follow-up/coordination	0.104 (0.032, 0.175)	0.005	0.142 (0.055, 0.228)	0.001

Note: *B* represents the unstandardized ordinary least squares regression coefficient. Both analyses used robust standard errors and were adjusted for age, sex, place of residence, marital status, number of chronic conditions, number of medications, ADL impairment, and depressive symptoms. The second analysis excluded participants with an EQ-5D-5L health utility value of 1.000 to assess the potential influence of upper-bound clustering. OLS, ordinary least squares; CI, confidence interval; PACIC, Patient Assessment of Chronic Illness Care; ADL, activities of daily living; EQ-5D-5L, EuroQol five-dimension five-level questionnaire.

Table S6. Exploratory sensitivity analysis using survey-site cluster-robust standard errors for the associations between PACIC scores and EQ-5D-5L health utility values.

Model	Variable	Average marginal effect (AME)	SE	*p* value	95% CI
Model 1: Overall PACIC score	PACIC total score	0.240	0.07	0.001	0.102, 0.378
Model 2: PACIC dimension scores	Patient activation	0.029	0.057	0.608	−0.083, 0.142
	Delivery system design/decision support	0.057	0.031	0.066	−0.004, 0.119
	Goal setting/tailoring	0.026	0.012	0.036	0.002, 0.049
	Problem solving/contextual counseling	0.087	0.019	< 0.001	0.049, 0.123
	Follow-up/coordination	0.094	0.022	< 0.001	0.050, 0.138

Note: Estimates were derived from two-limit Tobit regression models using cluster-robust standard errors with survey site as the clustering unit. Model 1 included the overall PACIC score, whereas Model 2 simultaneously included the five PACIC dimension scores. Both models were adjusted for age, sex, place of residence, marital status, number of chronic conditions, number of medications, ADL impairment, and depressive symptoms. AMEs represent the expected change in EQ-5D-5L health utility value associated with a one-point increase in the corresponding PACIC score. AME, average marginal effect; PACIC, Patient Assessment of Chronic Illness Care; ADL, activities of daily living; EQ-5D-5L, EuroQol five-dimension five-level questionnaire; CI, confidence interval.

Table S7. Sensitivity analysis excluding ADL impairment from covariate adjustment for the associations between PACIC scores and EQ-5D-5L health utility values

Model	Variable	Average marginal effect (AME)	SE	*p* value	95% CI
Model 1: Overall PACIC score	PACIC total score	0.271	0.057	< 0.001	0.160, 0.381
Model 2: PACIC dimension scores	Patient activation	0.035	0.033	0.290	-0.031, 0.102
	Delivery system design/decision support	0.066	0.037	0.071	-0.006, 0.139
	Goal setting/tailoring	0.026	0.033	0.430	-0.038, 0.090
	Problem solving/contextual counseling	0.096	0.031	0.002	0.036, 0.160
	Follow-up/coordination	0.092	0.041	0.023	0.013, 0.173

Note: Estimates were obtained from two-limit Tobit regression models after excluding ADL impairment from covariate adjustment. Model 1 included the overall PACIC score, whereas Model 2 simultaneously included the five PACIC dimension scores. Both models were adjusted for age, sex, place of residence, marital status, number of chronic conditions, number of medications, and depressive symptoms. AMEs represent the expected change in EQ-5D-5L health utility value associated with a one-point increase in the corresponding PACIC score. AME, average marginal effect; PACIC, Patient Assessment of Chronic Illness Care; ADL, activities of daily living; EQ-5D-5L, EuroQol five-dimension five-level questionnaire; CI, confidence interval.
